# Enhanced Electrical Properties of Copper Nitride Films Deposited via High Power Impulse Magnetron Sputtering

**DOI:** 10.3390/nano12162814

**Published:** 2022-08-16

**Authors:** Yin-Hung Chen, Pei-Ing Lee, Shikha Sakalley, Chao-Kuang Wen, Wei-Chun Cheng, Hui Sun, Sheng-Chi Chen

**Affiliations:** 1 Institute of Materials Science and Engineering, National Taiwan University, Taipei 106, Taiwan; 2 Department of Materials Engineering and Center for Plasma and Thin Film Technologies, Ming Chi University of Technology, New Taipei City 243, Taiwan; 3 Department of Mechanical Engineering, National Taiwan University of Science and Technology, Taipei 106, Taiwan; 4 School of Space Science and Physics, Shandong University, Weihai 264209, China; 5 College of Engineering and Center for Green Technology, Chang Gung University, Taoyuan 333, Taiwan

**Keywords:** copper nitride (Cu_3_N) thin films, High Power Impulse Magnetron Sputtering (HiPIMS), peak power density, deposition pressure, conductivity type

## Abstract

High Power Impulse Magnetron Sputtering (HiPIMS) has generated a great deal of interest by offering significant advantages such as high target ionization rate, high plasma density, and the smooth surface of the sputtered films. This study discusses the deposition of copper nitride thin films via HiPIMS at different deposition pressures and then examines the impact of the deposition pressure on the structural and electrical properties of Cu_3_N films. At low deposition pressure, Cu-rich Cu_3_N films were obtained, which results in the n-type semiconductor behavior of the films. When the deposition pressure is increased to above 15 mtorr, Cu_3_N phase forms, leading to a change in the conductivity type of the film from n-type to p-type. According to our analysis, the Cu_3_N film deposited at 15 mtorr shows p-type conduction with the lowest resistivity of 0.024 Ω·cm and the highest carrier concentration of 1.43 × 10^20^ cm^−3^. Furthermore, compared to the properties of Cu_3_N films deposited via conventional direct current magnetron sputtering (DCMS), the films deposited via HiPIMS show better conductivity due to the higher ionization rate of HiPIMS. These results enhance the potential of Cu_3_N films’ use in smart futuristic devices such as photodetection, photovoltaic absorbers, lithium-ion batteries, etc.

## 1. Introduction

Transition metal nitrides (TMNs) have been proved to be significant materials with a myriad of uses that hugely impact our daily lives. TMNs are highly advantageous in that they offer good hardness [1] and high temperature stability [2], resulting in them being highly recommended as a coating to protect mechanical tools [3]. Interestingly, many of these materials possess rock–salt structures [4], due to which they can either be metallic (like TiN [5]) or semiconductors (like ScN [6], YN [7] etc.). Among various TMNs with semiconductor properties, researchers in a quest for new kinds of materials possessing remarkable optoelectronic performance have put their key focus on Copper Nitride (Cu_3_N). Cu_3_N in crystalline form has an unambiguous cubic anti-ReO_3_ structure (a = 0.38 nm and α = β = γ = 90°) [8]. Optical band gap is temperature-dependent, lying within the range of 1.2 eV and 1.9 eV [9], and presenting less reflectivity [10] and high transparency in the IR region [11]. At room temperature, Cu_3_N with a lattice constant lower than 38 Å possesses huge electrical resistivity [10]. Thanks to abundant resource availability, cheap manufacturing costs, non-toxicity, low deposition temperature and also being highly adaptable to several substrates [12,13], Cu_3_N can be applied in numerous fields such as photodetectors [14], optical storage memory [15], integrated circuits [16], tunnel junctions [17], resistive random-access memory [18], solar energy conversion [19], photovoltaic absorber [20], diode rectifier [21], lithium-ion batteries [22], etc.

Recently, most of the attention has been focused on the formation of copper nitride films due to potential application in numerous fields. Fabrication of Cu_3_N using different techniques results in their physical properties being able to be controlled through certain manipulations of the parameters during the fabrication process. Thus far, several traditional techniques for the preparation of Cu_3_N have been studied in depth, including thermal oxidation [23], chemical vapor deposition [24], electrochemical lithium tuning [25], pulsed laser deposition [16], reactive RF sputtering [26], plasma-assisted molecular beam epitaxy [27], and DC magnetron sputtering [28]. Among these processes, magnetron sputtering has emerged as the most suitable process due to its tremendous uniformity across the deposited area, high reproducibility, rapid deposition, and decent controllability of the chemical composition [29]. However, its average degree of ionization of metallic vapor is lower than 10% [30]; in which the condition of the existing sputtered species are often neutral particles. With great advancement over the conventional magnetron sputtering technique, high power impulse magnetron sputtering (HiPIMS) technology has generated enormous interest recently as a result of the higher degree of plasma density as well as the ionization rate of the target installed. Additionally, HiPIMS provides high voltage and current during the deposition process and promotes a fast deposition rate. It can also be operated at low pressure and offers high sputtering rates, even during low duty cycle. Due to high incident target atomic energy, it increases the hardness and improves the material’s electrical properties, smoothness, and density with excellent adhesion [31,32,33,34,35]. According to our study, the ionization rate of Cu flux during deposition can achieve up to 70% in the HiPIMS process, which is significantly superior to that of the more common DC magnetron sputtering [36]. Although HiPIMS has many advantages in the synthesis of functional materials, few studies have concentrated on the formation of p-type nitride films using this technology. Besides this, sputtering atmosphere has also been confirmed as being critical on the film’s performance. Wang et al. [37] prepared Cu_3_N films while varying the nitrogen gas at different sputtering pressure and concluded that the bandgap of the films varies dependent upon the nitrogen gas pressure. In another study, Pierson [38] deposited Cu_3_N films using RF magnetron sputtering with Ar/N_2_ reactive mixtures, and the results show an increase in the lattice constant and the electrical resistivity of the film with the increased nitrogen flow rate in the chamber.

In this work, Cu_3_N thin films were deposited via HiPIMS technology at different deposition pressures while peak power density and the mixture of argon and nitrogen reactive gas remained constant. The impact of the deposition pressure on the optoelectronic properties of Cu_3_N thin films was then investigated. In our previous study [21] we used DCMS technology and changed the working pressures, and the results showed that an increase in substitution of Cu^2+^ ions for Cu^+^ ions will lead to the creation of Cu^+^ vacancies causing a transition in the conductivity type of the film from n-type to p-type. As HiPIMS has a higher ionization rate, it can generate more Cu^2+^ ions, resulting in the higher substitution of Cu^2+^ ions by replacing Cu^+^ ions. Moreover, upon increasing deposition pressure, more argon ions bombard the Cu target, which can sputter more Cu ions or atoms, so the probability of obtaining Cu^2+^ and Cu^+^ ionization states is high. It also enhances the transmittance of the film, whereas the reflection degrades. This is primarily due to the disappearance of the Cu-rich phase. Finally, the properties of the Cu_3_N thin films prepared via HiPIMS were compared to those of films deposited via conventional DCMS.

## 2. Materials and Methods

Cu_3_N thin films were deposited through high power impulse magnetron sputtering (HiPIMS) with a thickness of 100 nm on Corning Eagle XG glass and silicon substrates at room temperature. The substrates were 6.95 mm × 6.95 mm in size, whereas the diameter and the thickness of pure Cu metallic target are 50.8 mm and 6 mm. A constant power of 0.3 kW was supplied to the Cu target by SPIK 2000A pulse supply (Shen Chang Electric Co., New Taipei City, Taiwan). The base pressure of the chamber was maintained below 6.7 × 10^−4^ Pa prior to the deposition. The reactive gas mixture flow rate of argon and nitrogen was 30 sccm, totally. During the deposition of Cu_3_N thin films, the nitrogen flow ratio [N_2_/(Ar + N_2_)] of 60% was kept constant, while the deposition pressure was varied from 5 to 25 mtorr. Meanwhile, the pulse on-time (t_on_) was maintained as a constant of 50 μs and the pulse off-time (t_off_) was varied between 1050~1200 μs to obtain a peak power density of about 1000 W/cm^2^ (i.e., instantaneous voltage × instantaneous current/target area). The duty cycle can be calculated as t_on_/(t_on_ + t_off_).

A surface profilometer (Kosaka Surfcorder ET200, Tokyo, Japan) was employed to measure the thickness of the Cu_3_N thin films on glass. An X-ray diffractometer (XRD, Malvern PANalytical Empyrean, Malvern, UK) with Grazing Incidence X-ray Diffraction (GIXRD) using the Cu K_α_ radiation (the wavelength is 1.5406 Å) was used for the identification of the phase structure. Films’ compositions were analyzed using a JEOL JXA-8200 (Tokyo, Japan) electron probe X-ray microanalyzer (EPMA). X-ray photoelectron spectroscopy (XPS) using a Model Sigma Probe manufactured by Thermo VG Scientific Company (Waltham, MA, USA) was used to analyze the chemical states of the films. The films’ surface roughness was measured by atomic force microscope (AFM, Bruker Dimension Edge, Billerica, MA, USA). The microstructures of the films were investigated by high-resolution transmission electron microscopy (HR-TEM, JEOL JEM-2100). Hall effect analysis (AHM-800B, Agilent Technologies, Santa Clare, CA, USA) was employed for the films’ electrical properties. Finally, the films’ transmittance was measured by ultraviolet-visible (UV-Vis) spectrophotometer (Jasco-V-770, Tokyo, Japan).

## 3. Results

**Figure 1:**
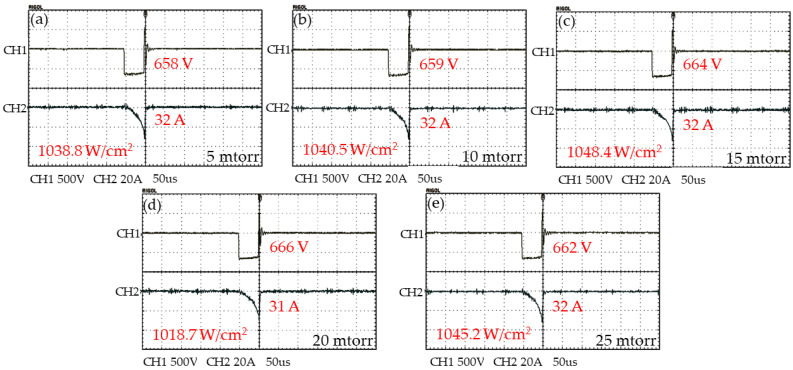
The instantaneous voltage and current during the HiPIMS deposition process with different deposition pressure (**a**) 5 mtorr, (**b**) 10 mtorr, (**c**) 15 mtorr, (**d**) 20 mtorr, and (**e**) 25 mtorr are represented by CH1 and CH2, respectively.

The instantaneous voltage and instantaneous current at varied deposition pressures were measured during the deposition process, as shown in Figure 1. The peak power density is numerically defined as the ratio of peak power to that of the target area. The value of peak power density at different deposition pressures remained above 1 kW/cm^2^. The instantaneous voltages and currents measured during the DCMS process are shown in Figure S1. The peak power density value is very different between DCMS (~0.015 kW/cm^2^) and HiPIMS (~1 kW/cm^2^), which reveals that HiPIMS has a higher ionization rate than DCMS.

**Figure 2:**
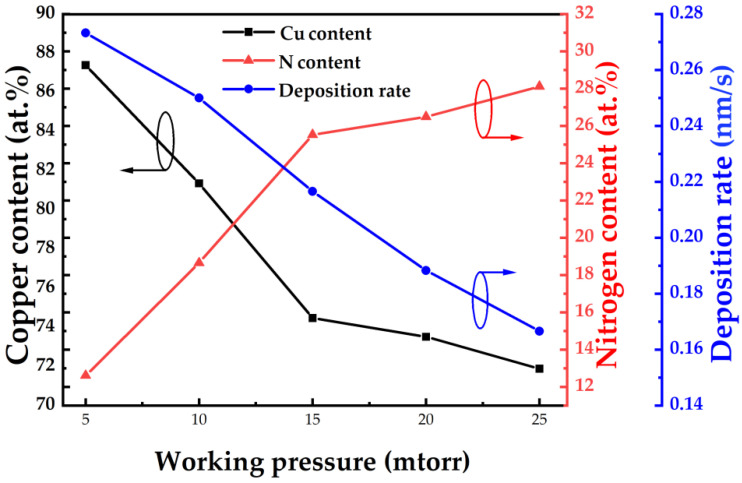
The analysis of the composition and deposition rate of Cu_3_N films deposited via HiPIMS at different deposition pressure.

The composition of Cu_3_N thin films produced via HiPIMS on silicon substrates at various deposition pressures is shown in Figure 2. Cu content reduces with increasing deposition pressure, while N content rises in the films. When the deposition pressure rises from 5 mtorr to 15 mtorr, Cu and N content suddenly changes. This is due to the fact that when the deposition pressure initially increases, more nitrogen molecules or atoms in the chamber react with copper atoms (ions), therefore the N content in the thin film increases. As the deposition pressure exceeds 15 mtorr, the variation in Cu and N content tends to moderate. Presumably, the composition of Cu_3_N film has reached saturation. Likewise, the deposition rate of the Cu_3_N thin film linearly declines with the increase in the deposition pressure. That is mainly due to the fact that as the amount of nitrogen and argon within the chamber rises, additional collisions among the particles will shorten their mean free path, which affects the deposition rate [21].

**Figure 3:**
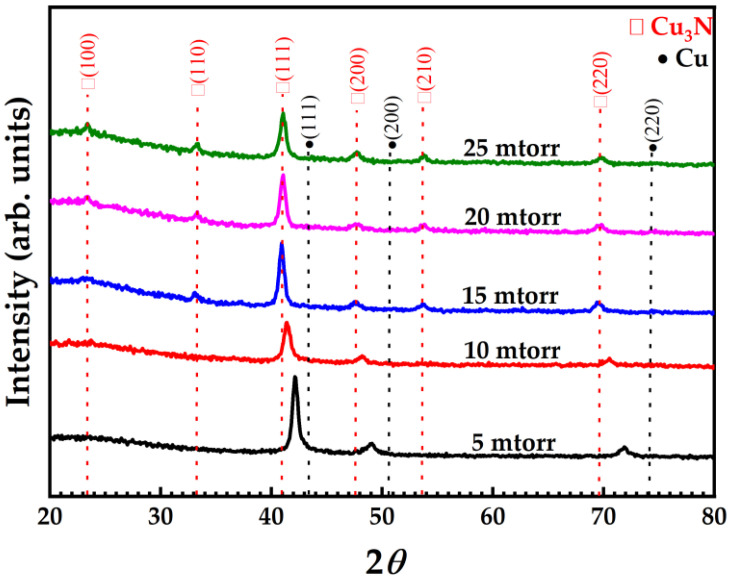
X-ray diffraction spectra of Cu_3_N films deposited via HiPIMS at different deposition pressure.

The X-ray diffractograms of Cu_3_N thin films (Figure 3) deposited on glass substrates at different deposition pressures mainly show diffraction peaks of the crystal planes of (100), (110), (111), (200), (210), and (220). The peaks corresponding to Cu_3_N phase are associated to JCPDS: 86-2283. The two most intense XRD peaks of Cu_3_N thin films are (111) and (100) related to copper and nitrogen content; usually considered as copper-rich and nitrogen-rich thin films, respectively [14]. Absence of impurity peaks in XRD results (like Cu) indicates the successful Cu nitration that helps in depositing Cu_3_N thin film with a high degree of purity. A diffraction peak of the Cu_3_N thin film deposited at a deposition pressure of 5 mtorr appears between the Cu (111) peak (JCPDF: 70-3038) and the Cu_3_N (111) peak (JCPDF: 86-2283). With the further increase in the deposition pressure, the diffraction peak (111) continues to move to lower Bragg angles until the deposition pressure exceeds 15 mtorr, which is completely matched with Cu_3_N, implying the development of pure Cu_3_N thin films. Although the (100) diffraction peak that produces the N-rich (100) plane does not appear as a result of insufficient nitrogen. Furthermore, there is a strong dominance of the (111) plane over the (100) plane in Cu_3_N at 10 mtorr, although in actuality, a small amount of the Cu_3_N (100) plane still exists, which is difficult to analyze by XRD. Additionally, the Cu_3_N (111) peak intensity declines with the rise in the deposition pressure. This may be the result of the decline in the copper content of the film (as observed in EPMA). It can be seen from some reports [37,39,40] that when the Cu content decrease, it will make it difficult to generate a Cu-rich (111) peak, thus resulting in a decrease in the intensity of the (111) peak.

**Figure 4:**
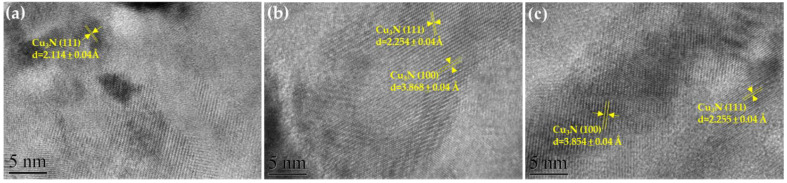
The HR-TEM images of Cu_3_N films deposited via HiPIMS at deposition pressure of (**a**) 10 mtorr, (**b**) 15 mtorr and (**c**) 20 mtorr. (For interpretation of the references to color in this figure’s legend, the reader is referred to the web version of this article).

The microstructure of the Cu_3_N nanocrystal on glass substrates was determined through HRTEM. The TEM sample was prepared through focused ion beam (FIB) for structural analysis. The HRTEM images of the cross-section of Cu_3_N thin films deposited at different deposition pressure were analyzed using Gatan Digital Micrograph software (Figure 4). After inverse Fourier transformation, the interplanar d-spacing value of the Cu_3_N thin-film lattice was calculated. Under low deposition pressure (10 mtorr) (Figure 4a), the film appears partially amorphous, with the d-spacing value as 2.114 ± 0.04 Å of the (111) plane. As the deposition pressure increased to 15 mtorr and 20 mtorr (Figure 4b,c) the d-spacing values of the (111) plane rise to 2.254 ± 0.04 Å and 2.255 ± 0.04 Å, respectively. Unlike the (111) plane, the d-spacing of the (100) plane in Figure 4b,c have similar value as 3.868 ± 0.04 Å and 3.854 ± 0.04 Å, respectively. From the XRD pattern (Figure 3), it is clear to see that when the deposition pressure rises from 10 mtorr to 20 mtorr, the Cu_3_N (111) diffraction peak progressively shifts to a lower angle, which means that the lattice constant gradually increases. This further confirms that the value of d-spacing increases with the increasing deposition pressure.

**Figure 5:**
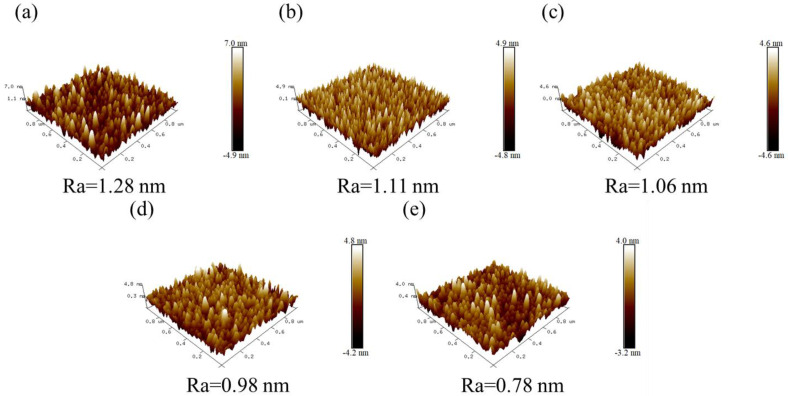
AFM images showing the surface (1 × 1 µm^2^) of Cu_3_N films deposited by HiPIMS via different deposition pressure (**a**) 5 mtorr, (**b**) 10 mtorr, (**c**) 15 mtorr, (**d**) 20 mtorr and (**e**) 25 mtorr.

The surface roughness of the deposited thin film plays an essential role in determining the film’s quality. This characteristic of Cu_3_N thin films on glass substrates (over an area of 1 µm × 1 µm) prepared by varying the deposition pressure was analyzed using atomic force microscopy (AFM, Figure 5). A significant reduction in the surface roughness of the Cu_3_N films with the increasing deposition pressure was observed. The value of the lowest surface roughness is Ra = 0.78 nm obtained at 25 mtorr. This corroborates that the smoothness of the films was improved with the increase in deposition pressure. When the deposition pressure increases, more Ar^+^ ions strike the copper target resulting in the high energy of the ion bombardment on the substrate due to the high peak power density of HiPIMS, which results in a close arrangement of the incident atoms in Cu_3_N films. In this way, the thin film with high deposition pressure achieved a smooth surface. In addition, the surface quality of the Cu_3_N film prepared via DCMS at 15 mtorr was also analyzed by AFM (as shown Figure S2). Its surface roughness (1 µm × 1 µm) Ra value of about 1.96 nm is higher than the roughness of the film deposited via HiPIMS (with Ra value of about 1.06 nm) (Figure 5c). During deposition using HiPIMS, the power density is much higher than that in the DCMS deposition process, as shown in (Figure 1c). As a result, more kinetic energy can be transferred from the bombarding Ar^+^ ions to the target atoms, which have Cu ions combined with nitrogen to the substrate, and result in a high- density film with a smooth surface. Therefore, the thin films via DCMS possessing low ionization have relatively rough surface.

**Figure 6:**
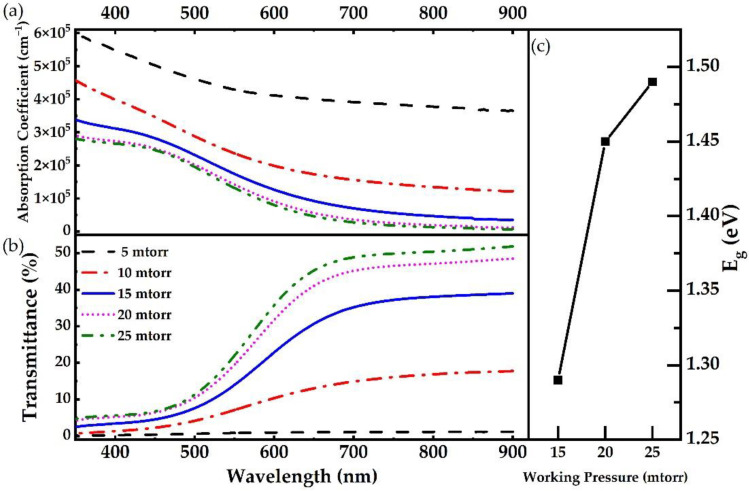
UV-Vis spectra showing optical properties (**a**) Absorption Coefficient, (**b**) Transmittance and (**c**) bandgap of Cu_3_N films deposited via HiPIMS at different deposition pressure.

The optical properties of Cu_3_N thin films deposited via HiPIMS on glass substrates were analyzed by UV-Vis spectroscopy (Figure 6a,b). The Cu_3_N thin films show high absorbance in the UV range (300–400 nm). As the wavelength increases, it starts to decrease gradually in the visible range from 400 nm to 700 nm. Beyond the visible region, it shows a low absorbance in the near IR range (>700 nm). Correspondingly, the Cu_3_N thin films show high transmittance in the IR range (700–900 nm), and as the wavelength decreases, it starts to decline gradually in the visible range from 700 nm to 450 nm wavelength. Below this region, it shows low transmittance in the near UV range (<400 nm). This behavior seems to be very common in the deposited Cu_3_N thin films. The deposition pressure drastically affects both the absorption coefficient and the transmittance of Cu_3_N films. When the deposition pressure changes, a significant variation in the absorption coefficient and the transmittance with the wavelength can be observed. Upon increasing the deposition pressure from 5 mtorr to 15 mtorr, there is an enormous decrease in the absorption coefficient and an impressive increase in transmittance. This drastic change is attributable to the significant decline in the Cu content in the films. Beyond 15 mtorr, the reduction in absorption coefficient and the transmittance seems to be weakened as a result of little variation in the film’s composition. The optical bandgap (*E_g_*) of the Cu_3_N can be calculated using the absorption coefficient. The Tauc method assumes that the energy-dependent absorption coefficient *α* can be expressed by the following equation:

*αh*ν = *A* (*h*ν – *E_g_*) ^x^ (1)

where *α* is the absorption coefficient, *h* is the Planck constant, ν is the photon frequency, *E_g_* is the bandgap, and *A* is a constant. The power of the x-factor depends on the nature of the electron transition, i.e., equal to 1/2 or 2 for the direct and indirect transition bandgap, respectively (Cu_3_N has the indirect transition bandgap, where x-factor is 2). Tauc plot of Cu_3_N representing the SI fitting as shown in Figure S3. Here, it is observed that with the rise in deposition pressure from 15 mtorr to 25 mtorr, the optical bandgap of p-type Cu_3_N also increases (Figure 6c).

**Figure 7:**
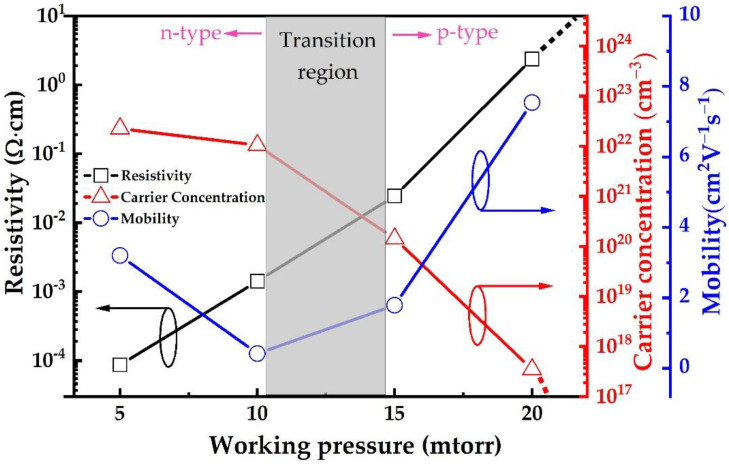
The electrical properties of Cu_3_N films deposited via HiPIMS at different deposition pressure.

Hall measurement was used to examine the electrical properties of the films deposited on glass substrates at various deposition pressure (Figure 7), while conductivity type was confirmed through hot probe measurement. Generally, the electrical properties depend upon the composition, phase structure and vacancy defects amount. By increasing the deposition pressure, the amount of nitrogen increases inside the reaction chamber. In low deposition pressure, the amount of nitrogen is inadequate to form Cu_3_N phase, which results in Cu-rich n-type Cu_3_N_x_ film. At 5 mtorr and 10 mtorr, the carrier concentration of n-type Cu_3_N films is found to be higher than 2.25 × 10^22^ cm^−3^ and 1.05 × 10^22^ cm^−3^, respectively. Their n-type conductivity occurs from the excessive electrons within Cu-rich films with low resistivity of 8.67 × 10^−5^ Ω·cm and 1.42 × 10^−3^ Ω·cm, respectively. Meanwhile, the mobility decreases from 3.2 to 0.42 cm^2^V^−1^s^−1^ with the increase in deposition pressure from 5 mtorr to 10 mtorr. Upon further increasing the deposition pressure, the N_2_ turns to be sufficient to form p-type Cu_3_N film by producing Cu^+^ vacancies. This conversion of the electrical conductivity type was observed by hot probe measurement at the deposition pressure of 15 mtorr, where the film’s resistivity and carrier concentration are 0.024 Ω·cm and 1.43 × 10^20^ cm^−3^, respectively. With increasing the deposition pressure, the resistivity increases and the carrier concentration decreases gradually. At the same time, the mobility increases from 1.79 to 7.54 cm^2^V^−1^s^−1^. After the deposition pressure exceeds 20 mtorr, the resistivity becomes very high to the point where it exceeds the measuring limit of the machine. This variation in the behavior of film’s electrical performance is consistent with the structural change of the films as observed from XRD data. The diffraction peak intensity becomes relatively weak as the deposition pressure increases to 20 mtorr and 25 mtorr, which is due to the change in the composition of Cu_3_N films. In addition, the electrical properties of Cu_3_N films deposited via HiPIMS were compared with those produced by other conventional methods, such as DCMS and RF sputtering (Table 1). Since p-type Cu_3_N film deposited via HiPIMS at 15 mtorr presents better electrical properties, as it possesses the lowest resistivity resulting from the high ionization rate offered by HiPIMS, we used the same parameters to deposit Cu_3_N film through DCMS. The results show that p-type Cu_3_N can be achieved, but its resistivity is above 18.82 Ω·cm (Table 1), which is excessive compared to that of the film deposited via HiPIMS.

**Figure 8:**
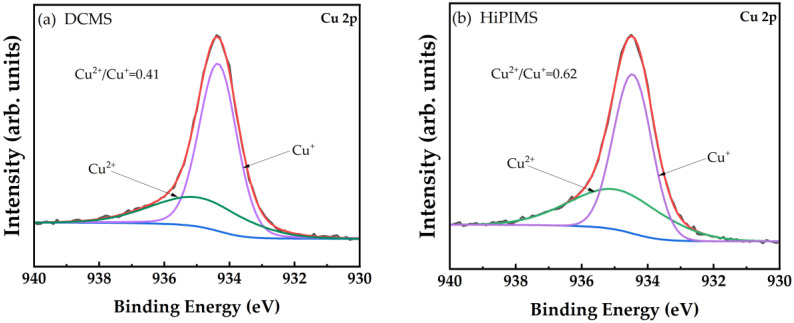
XPS spectra of Cu_2_p core levels of Cu_3_N films deposited using (**a**) DCMS and (**b**) HiPIMS modes. (For interpretation of the references to color in this figure legend, the reader is referred to the web version of this article).

The comparative investigation of the chemical bonding state in Cu_3_N thin films was also performed by X-ray photoelectron spectroscopy (XPS) to confirm the change in Cu^2+^ content within Cu_3_N thin films fabricated via both DCMS and HiPIMS technology on silicon substrates (Figure 8). The photoelectron emission from the Cu-2p spectra of Cu_3_N thin films was analyzed. The fitting curve of Cu-2p spectra was compared in Figure 8a,b. The Cu^2+^/Cu^+^ ratio of the film deposited via HiPIMS (0.62) is higher than that of the film deposited via DCMS (0.41), which indicates that a relatively high proportion of Cu^2+^ ions exist in the Cu_3_N thin films deposited via HiPIMS.

## 4. Discussion

Based on the conductivity type mechanism of NiO thin films, holes get generated by replacing two Ni^2+^ cations with two Ni^3+^ cations, which result in Ni^2+^ vacancies [48,49]. This theory suits the best as proof to describe the conductivity type transition mechanism of Cu_3_N (as shown in Figure S4). The above findings confirm that the replacement of Cu^+^ ions by Cu^2+^ ions are more evident in HiPIMS than that in DCMS, which results in more Cu^+^ vacancies during the deposition using HiPIMS process. It could contribute to higher p-type carrier concentration in the films deposited via HiPIMS technology, which helps in reducing the electrical resistivity of the films as compared to DCMS. These results clearly show the potential of Cu_3_N films deposited through HiPIMS technology to be applied in future optoelectronic devices. This technique helps in obtaining a flatter interface when the multilayer films are coated, which can improve the device performance [50].

## 5. Conclusions

Cu_3_N thin films were deposited via HiPIMS technology at different deposition pressures, while peak power density and the mixture of argon and nitrogen reactive gas remained constant. At lower deposition pressure, the existence of Cu-rich phase enhances the film’s n-type conductivity. Upon increasing the deposition pressure, the deposition rate and surface roughness decrease while nitrogen content in the film increases with the reduction in the copper content in the film. At the higher deposition pressures, the existence of a Cu_3_N phase is confirmed to increase the film’s transmittance and its optical bandgap, while also enhancing the resistivity of the Cu_3_N film. In addition, the study compares the chemical bonding state, quality, and electrical properties of Cu_3_N deposited through HiPIMS and DCMS methods. Beneficial due to its higher ionization rate and peak power density, HiPIMS offers lower roughness of the films and a higher Cu^2+^/Cu^+^ ratio, which results in more Cu^+^ vacancies. This indicates that HiPIMS technology is an ideal technology to achieve better electrical properties of Cu_3_N films in comparison to the transitional DCMS methods.

## Figures and Tables

**Table 1:** Comparison of the electrical properties of Cu_3_N films deposited by various sputtering techniques.

Material	Growth Method	Resistivity (Ω cm)	Carrier Concentration (cm^−3^)	Mobility (cm^2^V^−1^s^−1^)	Type	Year	Ref.
Cu_3_N	RF-Sputter	1.1 × 10^3^	-	-	n	2012	[41]
Cu_3_N	DC-Sputter	20	-	-	n	2011	[42]
Cu_3_N-(Zr, Cr)	RF-Sputter	1.65 × 10^−4^	-	-	n	2017	[43]
Cu_3_N-Mg	RF-Sputter	1	6.8 × 10^18^	6	n	2016	[44]
Cu_3_N-Pd	RF-Sputter	1.08 × 10^−3^	10^21^	18.9	n	2013	[45]
Cu_3_N	RF-Sputter	100	10^15^	1	p	2016	[46]
Cu_3_N	RF-Sputter	120	-	-	p	2015	[47]
Cu_3_N	DC-Sputter	0.33	1.29 × 10^19^	1.45	p	2019	[21]
Cu_3_N	DCMS	18.82	-	-	p	2022	This Study
Cu_3_N	HiPIMS	0.024	1.43 × 10^20^	1.79	p	2022	This Study

## Supplementary Materials

The following supporting information can be downloaded at: https://www.mdpi.com/article/10.3390/nano12162814/s1, Figure S1: The instantaneous voltage and current during the (a) HiPIMS and (b) DCMS process are represented by CH1 and CH2, respectively.; Figure S2: AFM images showing the surface (1 × 1 µm^2^) of Cu_3_N films deposited by DCMS at 15 mtorr.; Figure S3: Tauc plot of Cu_3_N representing the SI fitting.; Figure S4: The p-type conductivity mechanism of Cu_3_N.
