# Supplementary material for: Enhanced Electrical Properties of Copper Nitride Films Deposited via High Power Impulse Magnetron Sputtering
Source: Nanomaterials (Basel). 2022 Aug 16;12(16):2814. doi: 10.3390/nano12162814 (PMC9415204; doi:10.3390/nano12162814)
Supplement: Supplementary file 1 [file nanomaterials-12-02814-s001.zip › nanomaterials-1826083-supplementary.pdf]

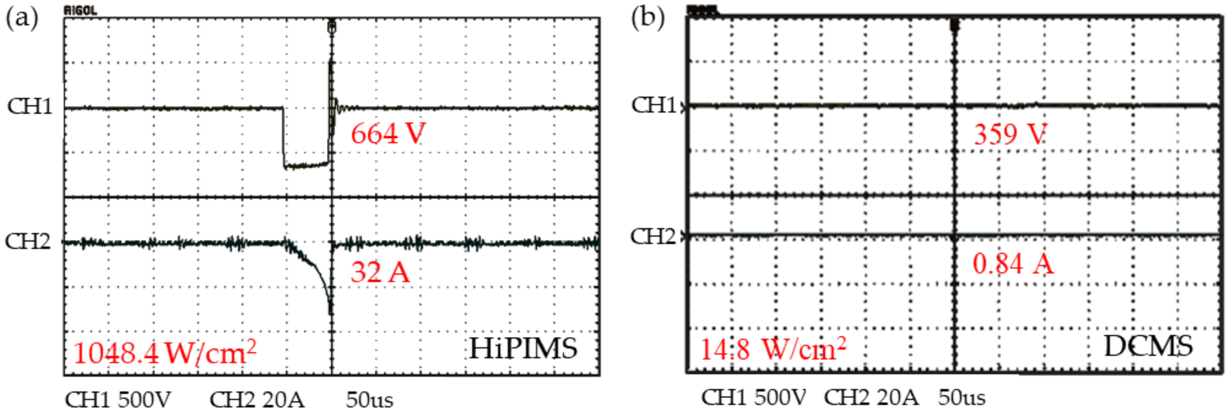

Figure S1. The instantaneous voltage and current during the (a) HiPIMS and (b) DCMS process are represented by CH1 and CH2, respectively.

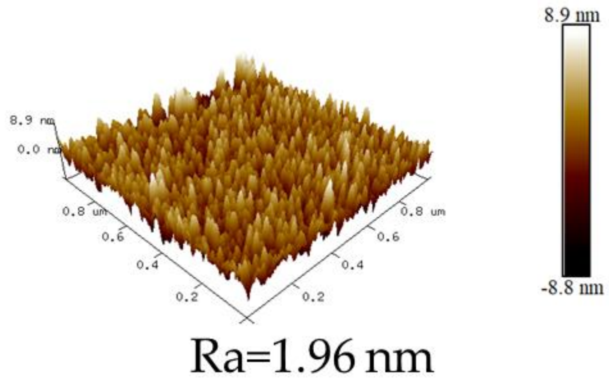

Figure S2. AFM images showing the surface (1×1 μm^2) of Cu_3N films deposited by DCMS at 15 mtorr.

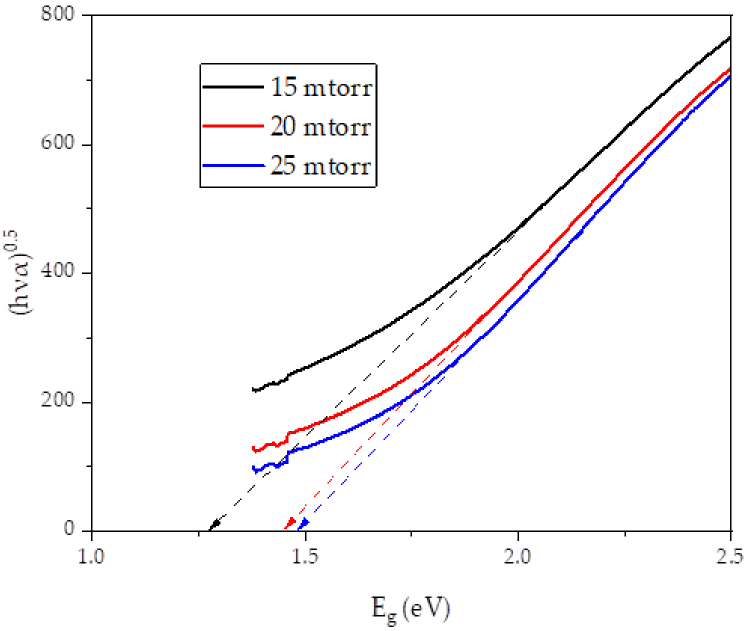

Figure S3. Tauc plot of Cu_3N representing the SI fitting

When substitution of Cu^{2+} ion for Cu^{+} ion, a Cu^{+} vacancy will appear, resulting in p-type conduction of the Cu_3N film.

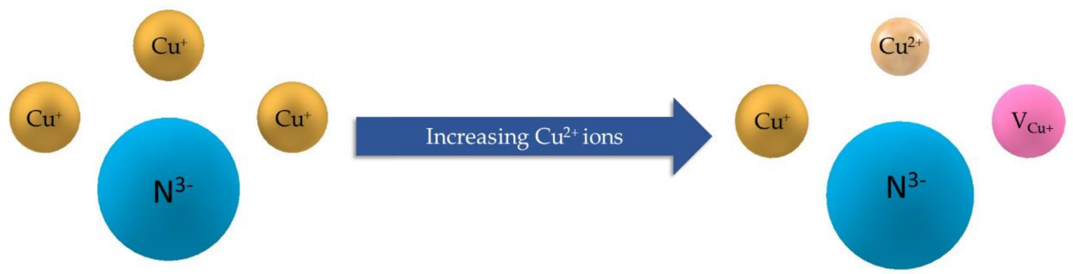

Figure S4. The p-type conductivity mechanism of Cu_3N.
